# Gate Metal‐Driven Sensing and Memory Bifunctionality in Intrinsically Stretchable Organic Electrochemical Transistors for Soft Neuromorphic Systems

**DOI:** 10.1002/advs.77505

**Published:** 2026-08-30

**Authors:** Jiyong Yoon, Jaepyo Jang, Hyunjin Jung, Duhwan Seong, Daeun Kim, Yewon Jeon, Ja Hoon Koo, Sang Min Won, Donghee Son

**Affiliations:** ^1^ Department of Electrical and Computer Engineering Sungkyunkwan University Suwon Republic of Korea; ^2^ Center for Neuroscience Imaging Research Institute For Basic Science Suwon Republic of Korea; ^3^ Department of Chemical and Biomolecular Engineering Yonsei University Seoul Republic of Korea; ^4^ Department of Chemistry Ajou University Suwon Republic of Korea; ^5^ Department of Semiconductor Systems Engineering Sejong University Seoul Republic of Korea; ^6^ Department of Intelligent Display Engineering Yonsei University Seoul Republic of Korea

**Keywords:** associative learning, bifunctional operation, organic electrochemical transistors, stretchable electronics

## Abstract

Intrinsically stretchable devices capable of integrating sensing, memory and signal processing are highly desirable for conformal bioelectronics. However, achieving such multifunctionality remains challenging due to conflicting operational requirements and the lack of mechanically compliant electrochemical gating strategies that preserve functional operation under deformation. Here, we present optimal materials and device strategies for intrinsically stretchable organic electrochemical transistors (OECTs) that enable gate metal‐driven bifunctionality within a single integrated platform. The device is constructed using a phase‐separated elastic semiconductor coupled with an ionically conductive alginate hydrogel, together with two stretchable gate electrodes: (1) Ag/AgCl for transient electrochemical responses; and (2) Au for persistent conductance modulation. By selectively addressing these gate electrodes, the OECT supports both fast, reversible responses for transient signal detection using bio‐inspired input waveforms, while also enabling stable conductance modulation for synapse‐like memory behavior, which is further utilized to implement artificial neural network (ANN)‐based pattern recognition using experimentally extracted conductance states. Furthermore, the bifunctional operation enables associative learning behavior in which repeated paired inputs progressively strengthen the device response, leading to successful threshold‐based decision making even under deformation up to 30% strain. Our work establishes an efficient methodology for achieving soft brain‐inspired OECT platforms that integrate sensing, memory and neuromorphic functionality.

## Graphical abstract

Intrinsically stretchable OECTs enable bifunctional operations via gate engineering. Polarizable Ag/AgCl gates provide volatile sensing, while Au gates enable non‐volatile memory. The SEBS‐blended channel maintains performance under strain, supporting reliable signal transduction. This platform advances soft bioelectronics integrating sensing and memory within a single mechanically compliant device architecture.

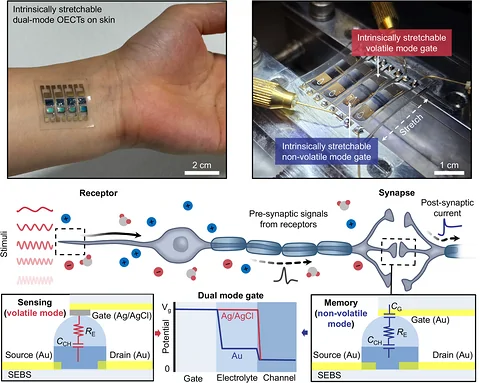

## 1 Introduction

Soft bioelectronics, based on intrinsically deformable and mechanically compliant materials, have emerged as promising platforms for biointegrated healthcare systems, including wearable sensors and electronic skins [1, 2, 3, 4], implantable neural and cardiac interfaces [5, 6, 7] and closed‐loop soft prostheses for sensorimotor function restoration [8, 9, 10, 11]. Their tissue‐like mechanical properties allow conformal contact with dynamic biological surfaces and support signal acquisition and feedback stimulation in mechanically deformable environments [12].

Recent advances in stretchable electrodes, tissue‐adhesive interfaces and self‐healing electronic materials have further expanded soft bioelectronics from passive physiological monitoring to active therapeutic intervention and functional restoration [13, 14, 15, 16, 17]. However, biological nervous systems do not simply record or stimulate signals, rather they continuously sense, memorize and adapt to temporally correlated stimuli through receptor–synapse networks [18, 19]. Therefore, next‐generation soft bioelectronics require neuromorphic functions that emulate sensory perception, synaptic plasticity and adaptive learning while maintaining mechanical compliance with biological tissues [20, 21, 22, 23, 24, 25, 26].

To realize such neuromorphic functions in soft and bio‐integrated formats, organic ionic–electronic materials provide a particularly attractive platform. In these materials, mobile ions can penetrate and accumulate within soft conjugated polymers through electrostatic coupling, redox reactions or ion‐assisted charge compensation, thereby directly converting ionic biological inputs into electronic outputs [27, 28, 29]. Moreover, the time‐dependent migration, trapping and relaxation of ions can generate history‐dependent conductance modulation, offering a materials‐level basis for short‐term dynamics and memory‐like behavior. Among organic ionic–electronic devices, organic electrochemical transistors (OECTs) are especially promising because they combine ion‐to‐electron transduction, low‐voltage operation and intrinsic signal amplification in a single transistor‐type architecture [30, 31, 32, 33, 34, 35]. In particular, OECT‐based synaptic transistors have been actively explored for neuromorphic computing, employing strategies such as gel‐electrolyte integration and molecular ordering to achieve tunable synaptic plasticity and multilevel conductance retention [36, 37].

Despite these advantages, most existing soft bioelectronic and organic neuromorphic systems still rely on spatially separated sensing, memory and processing components. Such architectures increase data‐transfer complexity, power consumption and latency during receptor–synapse‐like signal conversion [38, 39, 40, 41, 42, 43]. In addition, many organic devices operate primarily as passive sensing or memory elements, making it difficult to independently regulate volatile sensory responses and non‐volatile synaptic states within the same soft device platform. This limitation is particularly critical because sensory detection requires rapid and reversible ion relaxation, whereas synaptic memory requires stable ion retention and conductance modulation. Moreover, although volatile/non‐volatile switching has been demonstrated through gate or ion‐dynamics engineering, such bifunctionality has so far been realized only in rigid devices, whereas intrinsically stretchable OECTs have typically imparted strain at the channel level while relying on externally supplied, non‐stretchable gate electrodes and realizing only a single operation mode (Table S1). A platform that combines full‐component stretchability with switchable volatile/non‐volatile operation has thus remained elusive. Therefore, an intrinsically stretchable OECT platform that integrates sensing, memory and neuromorphic processing within a single device architecture is needed to realize in‐sensor bioelectronic computation [44, 45].

**FIGURE 1:**
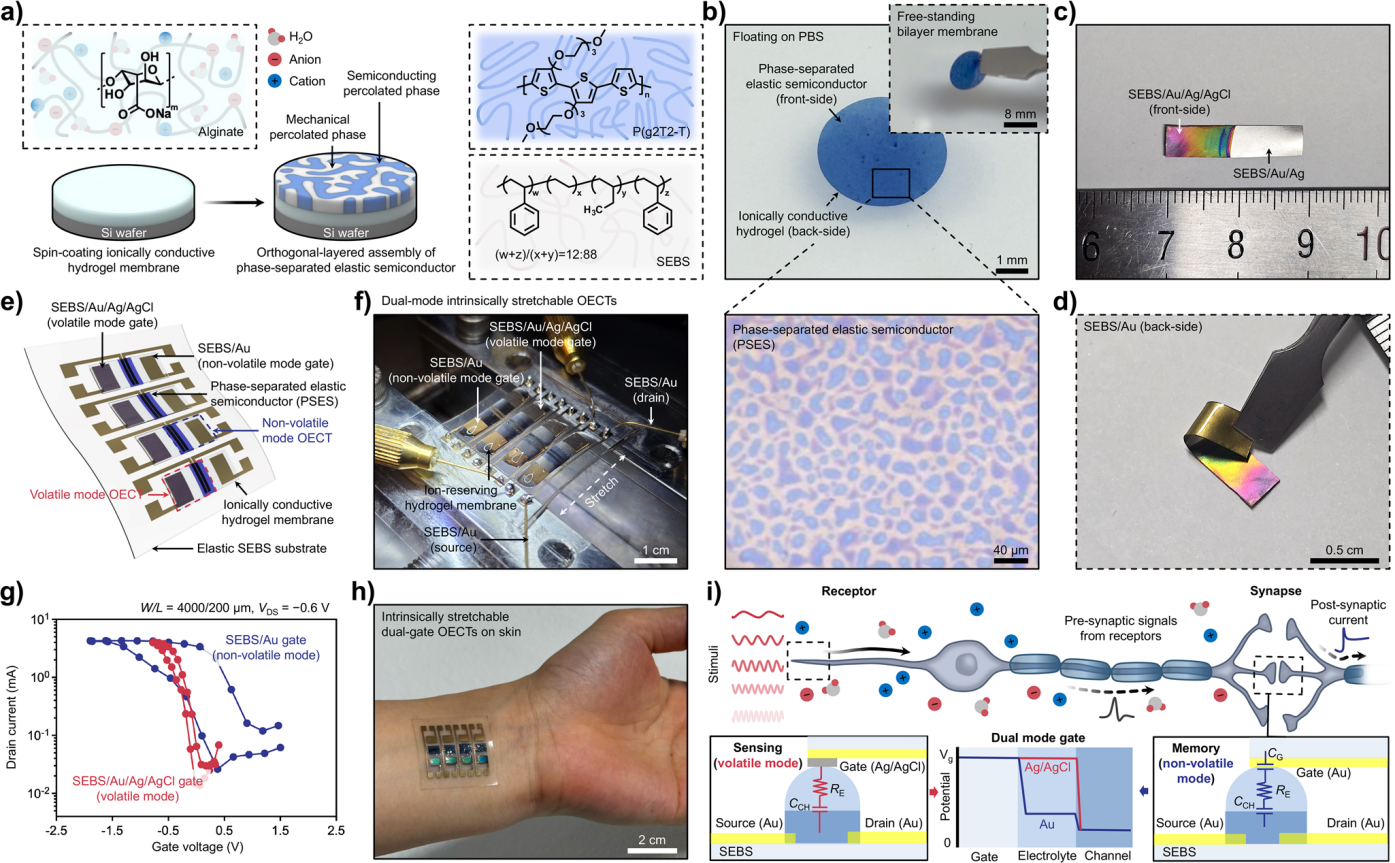
Dual‐gate intrinsically stretchable OECTs based on PSES. (a) Schematic of the fabrication of an ionically conductive hydrogel/PSES bilayer membrane. An ionically conductive alginate hydrogel membrane is spin‐coated on a Si wafer, followed by orthogonal‐layer assembly of a P(g2T2‐T):SEBS (7:3) elastic semiconductor, forming bi‐continuous semiconducting and mechanically percolated phases. The chemical structures of alginate, P(g2T2‐T), and SEBS are shown. (b) Photographs of the free‐standing bilayer membrane floating on PBS, showing the PSES front side and the ionically conductive hydrogel back side. The inset shows handling of the free‐standing bilayer membrane. The magnified optical microscopy image shows the phase‐separated morphology of the elastic semiconductor (bottom dashed box). (c,d) Photographs of the front side of the intrinsically stretchable SEBS/Au/Ag/AgCl electrode, showing the Ag/AgCl region formed on the Ag‐coated SEBS/Au electrode (c) and the back side of the SEBS/Au electrode, highlighting its crack‐based intrinsically stretchable nature (d). (e) Schematic of the intrinsically stretchable dual‐gate OECTs. Each transistor channel is integrated with two different gate electrodes, consisting of an Au gate for non‐volatile mode operation and an Ag/AgCl gate for volatile mode operation. The PSES serves as the channel, and the ionically conductive hydrogel membrane acts as the electrolyte reservoir. (f) Photograph of the intrinsically stretchable dual‐gate OECTs under stretching, showing the source/drain electrodes together with the Au non‐volatile gate and Ag/AgCl volatile gate integrated for each channel. (g) Representative transfer characteristics of the intrinsically stretchable dual‐gate OECTs measured using the Au gate (blue, non‐volatile mode) and Ag/AgCl gate (red, volatile mode). (h) Photograph of the intrinsically stretchable dual‐gate OECTs laminated on human skin, demonstrating conformal and wearable integration. (i) Schematic of gate‐interface‐dependent bifunctional operation. The quasi‐non‐polarizable Ag/AgCl gate minimizes the gate‐side potential drop, enabling reversible ionic modulation and volatile sensing. In contrast, the finite interfacial capacitance of the polarizable Au gate causes additional voltage division and, together with partial ion retention in the PSES channel, enables non‐volatile memory. *C*_G_, gate/electrolyte interfacial capacitance; *R*_E_, electrolyte resistance; *C*_CH_, channel capacitance.

Here, we report an intrinsically stretchable dual‐gate OECT that integrates receptor‐like volatile sensing and synapse‐like non‐volatile memory within a single mechanically compliant device. The device combines an ionically conductive alginate hydrogel membrane, a phase‐separated poly[3,3*'*‐bis[2‐[2‐(2‐methoxyethoxy)ethoxy]ethoxy]‐2,2*'*:5*'*,2*''*‐terthiophene‐5,5*''*‐diyl] (P(g2T2‐T)):polystyrene‐*block*‐poly(ethylene‐*ran*‐butylene)‐*block*‐polystyrene (SEBS) elastic semiconductor (PSES) and two electrochemically distinct stretchable gate electrodes. In this architecture, all functional components are intrinsically stretchable and co‐integrated, in contrast to previous stretchable OECTs in which the gate electrode is supplied externally. The ionically conductive hydrogel membrane provides soft electrolyte coupling, whereas the elastic semiconductor forms a deformable mixed ionic–electronic channel for transistor operation (Figure 1a,b). The SEBS/Au/Ag/AgCl gate enables reversible volatile electrochemical gating for sensory transduction, while the SEBS/Au gate induces persistent conductance modulation for synaptic memory (Figure 1c–g). By assigning volatile and non‐volatile operation to two gate interfaces within a single stretchable transistor, the device achieves gate‐dependent switching between volatile sensing and non‐volatile memory modes. This dual‐gate platform allows co‐localized signal transduction and storage, providing the basis for sensing, synaptic plasticity, and Pavlovian associative learning with threshold‐based adaptive responses under mechanical deformation (Figure 1h,i) [46, 47].

## 2 Results and Discussion

### 2.1 Design and Fabrication of Intrinsically Stretchable Dual‐Gate OECTs

To realize a mechanically compliant ion–electronic platform capable of co‐localized volatile sensing and non‐volatile memory, we designed an intrinsically stretchable dual‐gate OECT based on an ionically conductive hydrogel/elastic semiconductor bilayer membrane (Figure 1a). The bilayer membrane was used as a transferable active channel, allowing the soft ion–electronic layer to be integrated onto pre‐patterned SEBS/Au source–drain electrodes without directly processing the hydrogel on the device substrate. The ionically conductive hydrogel layer provides an internal electrolyte reservoir after hydration, whereas the PSES serves as a deformable mixed ionic–electronic channel for OECT operation.

A key requirement for this architecture is maintaining the structural integrity of the hydrogel‐containing membrane during hydration and transfer. Hydrated ionically conductive hydrogels can swell, soften and partially dissociate in aqueous environments, which can make free‐standing handling difficult [12]. To overcome this limitation, SEBS was incorporated into the P(g2T2‐T) semiconductor layer to form an elastic semiconductor interface. As a result, the SEBS‐reinforced bilayer membrane retained its macroscopic shape while floating on PBS and could be transfer‐laminated onto the pre‐patterned SEBS/Au source–drain electrodes to define the OECT channel with stable handling and precise placement (Figure 1b, top).

The structural stability of the bilayer membrane is supported by the phase‐separated morphology of the P(g2T2‐T):SEBS elastic semiconductor (Figure 1b, bottom). The elastic semiconductor exhibited interconnected semiconductor‐rich and SEBS‐rich domains, indicating the formation of a bi‐continuous composite rather than a homogeneous brittle semiconductor layer on hydrogel membrane (Figures S1–S4). In this morphology, the P(g2T2‐T)‐rich domains provide continuous mixed ionic–electronic transport pathways, while the SEBS‐rich matrix preserves mechanical continuity during swelling, transfer and stretching. Consistently, P(g2T2‐T)/hydrogel bilayer membrane‐based devices showed strain‐induced current degradation and mechanical failure, confirming that the pure semiconductor channel cannot support stretchable dual‐gate OECT operation (Figure S5). This PSES therefore enables the active channel to function simultaneously as an ion–electronic conductor and as a mechanically robust component of the free‐standing bilayer membrane (Figure S6).

Using this transferable bilayer channel, we fabricated a dual‐gate OECT by integrating two electrochemically distinct stretchable gate electrodes on a homogeneous SEBS‐based platform (Figure S7). The SEBS/Au/Ag/AgCl gate provides a stable and reversible ionic interface for volatile electrochemical gating, whereas the SEBS/Au gate induces persistent conductance modulation for non‐volatile synaptic memory. The Ag/AgCl layer was formed directly on the intrinsically stretchable SEBS/Au electrode [4, 48, 49, 50], enabling the volatile gate to maintain mechanical deformability while functioning as an electrochemically stable gate interface (Figure 1c,d). The resulting device integrates the ionically conductive bilayer channel, SEBS/Au source–drain electrodes, SEBS/Au/Ag/AgCl volatile gate and SEBS/Au non‐volatile gate into a single mechanically stretchable OECT architecture (Figure 1e and Figure S7). This fully integrated dual‐gate OECT could undergo tensile deformation while retaining gate‐controlled transistor operation, demonstrating the compatibility of the dual‐gate configuration with mechanically stretchable operation (Figure 1f,g). In particular, the SEBS/Au/Ag/AgCl gate produced reversible transfer characteristics with a narrow hysteresis window, whereas the SEBS/Au gate generated pronounced hysteresis, confirming volatile and non‐volatile operation modes in the same stretchable OECT channel.

The intrinsically stretchable dual‐gate OECTs could also be laminated onto human skin, demonstrating conformal and wearable integration of the device platform (Figure 1h). This skin‐mounted configuration highlights the mechanical compliance of the SEBS‐based device architecture and its potential application to soft bioelectronic interfaces. Conceptually, the dual‐gate OECT is designed to emulate receptor–synapse coupling in biological nervous systems (Figure 1i). In this framework, the quasi‐non‐polarizable Ag/AgCl gate enables reversible volatile sensing, whereas the finite interfacial capacitance of the polarizable Au gate, together with partial ion retention in the PSES channel, enables non‐volatile conductance modulation analogous to synaptic memory. Therefore, the dual‐gate architecture provides a single stretchable transistor platform for co‐localizing sensory transduction and memory formation through two electrochemically distinct gate interfaces, establishing the device basis for receptor–synapse‐integrated neuromorphic operation.

### 2.2 Characterization of Intrinsically Stretchable Ag/AgCl Gate Electrodes and PSES

For bifunctional OECT operation, the two gate electrodes must provide electrochemically distinct gating interfaces within a mechanically deformable device architecture. In particular, the volatile gate should enable reversible ionic coupling to the elastic semiconductor channel without inducing large interfacial polarization or irreversible conductance changes. Ag/AgCl materials are widely used as stable electrochemical interfaces because the reversible Ag/AgCl redox couple can effectively exchange ionic charge with chloride‐containing electrolytes. However, conventional Ag/AgCl electrodes are mechanically rigid and therefore incompatible with intrinsically stretchable OECT platforms [48, 51]. To address this limitation, we developed an intrinsically stretchable SEBS/Au/Ag/AgCl electrode by forming an Ag/AgCl layer directly on a crack‐based SEBS/Au stretchable electrode. This design allows the Ag/AgCl surface to function as an electrochemically stable volatile gate, while the underlying Au network embedded in the SEBS elastomer maintains electrical conductivity during mechanical deformation.

**FIGURE 2:**
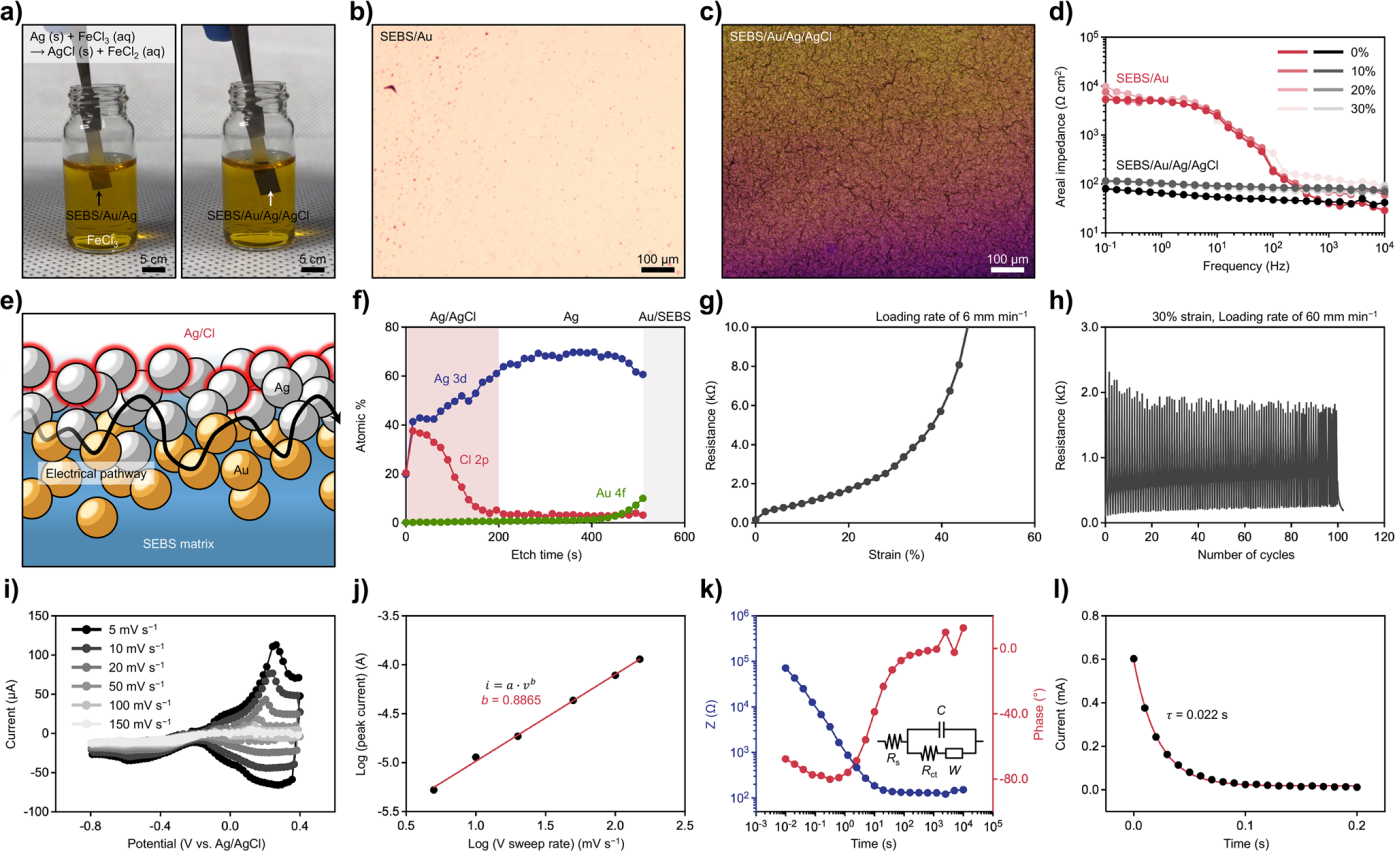
Characterization of intrinsically stretchable SEBS/Au/Ag/AgCl electrodes and PSES. (a) Photographs showing the chemical chlorination of Ag deposited on SEBS/Au electrodes in FeCl_3_ solution to form AgCl, according to the reaction Ag(s) + FeCl_3_(aq) → AgCl(s) + FeCl_2_(aq). (b,c) Optical microscopy images of the SEBS/Au electrode surface (b) and the SEBS/Au/Ag/AgCl electrode surface after chemical chlorination, showing the formation of a rough Ag/AgCl layer on the intrinsically stretchable SEBS/Au electrode (c). (d) Areal impedance spectra of SEBS/Au and SEBS/Au/Ag/AgCl electrodes under tensile strains of 0%, 10%, 20%, and 30%. (e) Schematic of the SEBS/Au/Ag/AgCl electrode structure, in which the Ag/AgCl layer provides an electrochemically active interface while the underlying Au network embedded in the SEBS matrix preserves electrical pathways under mechanical deformation. (f) XPS depth profile of the SEBS/Au/Ag/AgCl electrode, showing the AgCl‐rich surface layer, underlying Ag layer, and buried Au/SEBS region. The distributions of Ag 3d, Cl 2p, and Au 4f confirm the formation of AgCl on Ag‐coated SEBS/Au. (g) Resistance change of the SEBS/Au/Ag/AgCl electrode under uniaxial tensile strain at a loading rate of 6 mm min^−^^1^. (h) Resistance response of the SEBS/Au/Ag/AgCl electrode during repeated 30% tensile stretching–releasing cycles at a loading rate of 60 mm min^−^^1^, demonstrating reproducible electrical continuity during cyclic mechanical deformation. (i) CV of the PSES measured at different scan rates from 5 to 150 mV s^−^^1^, showing reversible electrochemical doping and dedoping behavior. (j) Logarithmic plot of the peak current as a function of voltage sweep rate. The power‐law fitting gives a b‐value, indicating predominantly surface‐confined/capacitive charge‐storage behavior with partial diffusion contribution in the PSES. (k) EIS impedance spectra of the PSES, showing the frequency‐dependent impedance modulus and phase response. (l) Transient current response of the PSES after voltage biasing, showing rapid current relaxation with a time constant of *τ*, indicating fast ion–electron coupling dynamics in the elastic semiconductor channel.

The SEBS/Au/Ag/AgCl electrode was fabricated by chemically chlorinating an Ag‐coated SEBS/Au electrode in FeCl_3_ solution (Figure 2a and Figure S8). The Ag‐coated electrode was immersed in the FeCl_3_ solution, where Ag was converted into AgCl through the reaction Ag(s) + FeCl_3_(aq) → AgCl(s) + FeCl_2_(aq). This simple wet‐chemical chlorination process produced a visible color change on the electrode surface, indicating the formation of AgCl on the Ag layer. Optical microscopy images further confirmed the surface transformation after chlorination. Before chlorination, the SEBS/Au electrode showed a relatively smooth metallic surface, whereas Ag/AgCl formation produced a rough and granular surface on the intrinsically stretchable electrode (Figure 2b,c). This roughened morphology is expected to increase the effective electrochemical surface area and facilitate ionic charge exchange at the electrode/electrolyte interface. The electrochemical advantage of the Ag/AgCl surface was evaluated by electrochemical impedance spectroscopy under tensile deformation (Figure 2d). Compared with the SEBS/Au electrode, the SEBS/Au/Ag/AgCl electrode exhibited a consistently lower impedance over the measured frequency range. The impedance reduction was particularly pronounced in the low‐frequency region, where electrode polarization typically dominates the interfacial response. This result indicates that the Ag/AgCl layer forms a more non‐polarizable and electrochemically efficient interface than bare Au. Importantly, this low‐impedance response was maintained under tensile strains of 0, 10%, 20%, and 30%, indicating that the Ag/AgCl layer provides an electrochemically efficient gate/electrolyte interface while the underlying SEBS/Au electrode preserves lateral electronic conduction during deformation. Such low interfacial impedance is important for volatile OECT gating because it allows the applied gate bias to be effectively coupled to the ionically conductive hydrogel/elastic semiconductor channel, minimizing voltage loss at the gate/electrolyte interface.

To further clarify the role of the Au interlayer, we also compared the strain‐dependent electrochemical impedance spectroscopy (EIS) response with an Au‐free SEBS/Ag/AgCl control electrode (Figure S9). Although the Au‐free SEBS/Ag/AgCl electrode showed an Ag/AgCl‐like low‐impedance response in the unstrained state, its impedance increased markedly under tensile deformation. This comparison indicates that direct Ag/AgCl formation on SEBS can provide an electrochemical interface but lacks a mechanically stable current‐collecting pathway under strain. Therefore, the Au interlayer does not deteriorate the stretchable Ag/AgCl gate; rather, the microcracked SEBS/Au layer stabilizes the electronic conduction pathway beneath the Ag/AgCl surface during mechanical deformation.

The structural design of the stretchable Ag/AgCl gate is schematically illustrated in Figure 2e. The top Ag/AgCl layer serves as the electrochemically active interface for reversible ionic exchange, whereas the underlying Au layers provide percolative conduction pathways [4]. Importantly, the Au conductive network is embedded in the SEBS elastomer matrix, allowing the Ag/AgCl electrode to accommodate tensile deformation through the crack‐based stretchable architecture. Therefore, the SEBS/Au/Ag/AgCl electrode combines two functions that are typically difficult to integrate: (1) stable electrochemical gating and (2) intrinsic mechanical stretchability. The depth‐dependent composition of the electrode was analyzed by X‐ray photoelectron spectroscopy (XPS) depth profiling to confirm the formation and vertical distribution of the Ag/AgCl layer (Figure 2f). The Cl 2p signal was dominant near the surface together with the Ag 3d signal, indicating the presence of an AgCl‐rich surface layer. With increasing etching depth, the Cl 2p signal decreased while the Ag 3d signal remained, suggesting the transition from the AgCl‐containing surface to the underlying Ag layer. At greater depths, the Au 4f signal became more pronounced, corresponding to the buried Au layer in the SEBS/Au electrode. These depth‐dependent elemental distributions confirm that chemical chlorination selectively formed an AgCl‐rich layer on the Ag‐coated SEBS/Au electrode without disrupting the underlying stretchable Au conductive network.

The electromechanical behavior of the SEBS/Au/Ag/AgCl electrode was then evaluated under tensile deformation. The electrode resistance increased progressively with uniaxial strain, while electrical continuity was maintained throughout the tested strain range (Figure 2g). Under repeated 30% stretching–releasing cycles, the electrode exhibited a reproducible resistance response without open‐circuit failure (Figure 2h). The strain‐dependent resistance increase reflects the progressive opening of crack‐mediated conduction pathways. Nevertheless, the absence of open‐circuit failure and the reproducible resistance response during cyclic deformation confirm mechanically compliant electrical continuity rather than strain‐invariant conductivity.

To impart mechanical stretchability while preserving electrochemical functionality, SEBS was blended with the semiconducting polymer P(g2T2‐T) to form a composite channel layer. Elastomer incorporation is generally associated with disrupted charge transport pathways or increased trap density [52]. To systematically investigate this effect, the scan rate dependence of cyclic voltammetry (CV) responses was analyzed (Figures 2i,j and Figure S10a,b). The peak current (*i*) scales with scan rate (*v*) according to a power‐law relationship, *i = a·v^b^*, where *b* reflects the dominant charge storage mechanism [34]. A *b*‐value approaching 1 indicates capacitive‐dominated behavior, whereas *b ≈ 0.5* suggests diffusion‐limited processes. Both pure P(g2T2‐T) and the PSES exhibited *b*‐value close to unity (0.8956 and 0.8865, respectively), indicating predominantly capacitive charge storage with no pronounced shift toward diffusion‐limited behavior upon SEBS incorporation. Electrochemical impedance spectroscopy further supports this interpretation (Figure 2k and Figure S10c). At low frequencies (1 Hz), the pure P(g2T2‐T) film exhibited slightly lower impedance (915 Ω) than the PSES (1076 Ω), and both systems were well described by an equivalent circuit model (Figures S10d and S11 and Table S2). The Warburg element, associated with diffusion‐related impedance, exhibited only a marginal increase in the PSES, suggesting that diffusion limitations are not significantly exacerbated. In contrast, the capacitance of the PSES decreased relative to that of pure P(g2T2‐T), indicating that morphological changes induced by SEBS incorporation reduce the effective ion‐accessible area and effective electrochemically active volume. Furthermore, to probe dynamic ion transport and relaxation behavior, chronoamperometric measurements were performed under a −0.4 V pulse (Figure 2l and Figure S10e). The extracted time constants were 27.2 ms for pure P(g2T2‐T) and 21.8 ms for the PSES. The comparable, and slightly faster, response of the PSES indicates that SEBS incorporation does not degrade the dynamic response and may even slightly facilitate ion relaxation dynamics. Taken together, these results demonstrate that blending SEBS into the semiconducting channel enhances mechanical compliance without fundamentally perturbing the ion–electron coupling mechanism governing OECT operation, while maintaining comparable electrochemical and electrical characteristics.

### 2.3 Bifunctional Operation of Stretchable Dual‐Gate OECT

In bioelectronic systems, the operational mode of transistors plays a critical role in determining their suitability for specific applications. Non‐volatile behavior, characterized by persistent conductance states after removal of the input stimulus, is advantageous for memory storage and neuromorphic computing, where retention of past signals is required. In contrast, volatile operation, in which the device rapidly returns to its baseline state, is more desirable for real‐time biosignal acquisition, as it enables continuous tracking of dynamic physiological signals without signal accumulation or drift. Such fast recovery is particularly important for accurately resolving low‐amplitude and time‐varying bioelectrical signals, including electrophysiological activity.

The gate‐dependent transition between volatile and non‐volatile operation originates from the distinct electrochemical boundary conditions and voltage distributions at the gate/electrolyte interfaces. The reversible Ag/AgCl redox couple provides a quasi‐non‐polarizable interface, minimizing the gate‐side potential drop and enabling reversible ion injection and extraction. In contrast, the polarizable Au gate introduces a finite interfacial capacitance and additional voltage division. Together with partial ion retention in the PSES channel, this produces pronounced hysteresis and persistent conductance modulation [53, 54] (Figure 1i).

**FIGURE 3:**
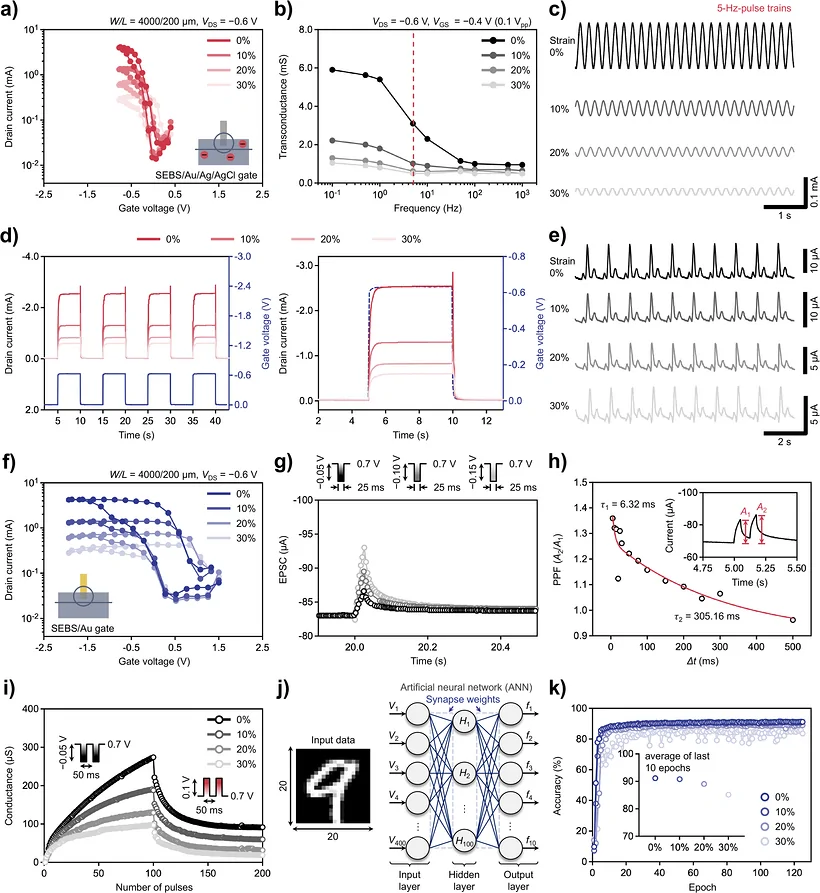
Bifunctional operation of intrinsically stretchable dual‐gate OECTs. (a) Transfer characteristics of the SEBS/Au/Ag/AgCl gate OECT operated in volatile mode under tensile strains of 0%, 10%, 20,% and 30%. The device retains gate‐controlled modulation with strain‐dependent attenuation under mechanical deformation. (b,c) Frequency‐dependent transconductance of the volatile‐mode OECT under tensile strains of 0%, 10%, 20%, and 30%, measured at *V*_DS_ = −0.6 V and *V*_GS_ = −0.4 V with a sinusoidal gate input of 0.1 V_pp_ (b) and dynamic current responses of the volatile‐mode OECT to 5‐Hz gate‐pulse trains under tensile strains of 0%, 10%, 20%, and 30%, showing stable pulse‐resolved operation under mechanical deformation (c). (d) Drain‐current responses of the volatile‐mode OECT to repeated gate‐voltage pulses under tensile strains of 0%, 10%, 20%, and 30% (left). The magnified response shows strain‐dependent current modulation while preserving reversible volatile switching behavior (right). (e) Volatile‐mode OECT responses to ECG‐like input signals under tensile strains of 0%, 10%, 20%, and 30%, demonstrating the capability to process physiologically relevant cardiac waveforms under mechanical deformation. (f) Transfer characteristics of the SEBS/Au gate OECT operated in non‐volatile mode under tensile strains of 0%, 10%, 20%, and 30%. The pronounced hysteresis reflects gate‐induced non‐volatile modulation of the channel conductance, which remains observable under stretching, although the current level and loop characteristics change with strain. (g) Excitatory post‐synaptic current (EPSC) of the non‐volatile‐mode OECT triggered by single voltage spikes with different amplitudes. The single pulse stimulation was applied to the SEBS/Au gate, and the resulting EPSC was recorded from the channel. (h) Paired‐pulse facilitation (PPF) index (*A*_2_/*A*_1_) as a function of the inter‐spike interval (*Δt*). The PPF behavior is fitted, showing fast and slow ionic relaxation with time constants of *τ*_1_ and *τ*_2_. The inset defines *A*_1_ and *A*_2_ from two sequential EPSC peaks at *Δt* = 75 ms. (i) Conductance modulation of the non‐volatile‐mode OECT under repeated potentiation and depression pulses at tensile strains of 0%, 10%, 20%, and 30%. The device exhibits gradual conductance increase and decrease in response to sequential gate pulses. (j) Schematic of an artificial neural network implemented using the experimentally measured conductance states of the intrinsically stretchable non‐volatile‐mode OECTs. The network consists of 400 input layers, 100 hidden layers, and 10 output layers for handwritten digit recognition. (k) Recognition accuracy of the artificial neural network as a function of epoch using conductance modulation characteristics measured under 0%, 10%, 20%, and 30% strain. The accuracy decreases gradually with increasing strain, reflecting the reduced number of effective conductance states, while remaining above 85% even at 30% strain, indicating functional neuromorphic operation under mechanical deformation.

The electromechanical behavior of the devices was evaluated under uniaxial strain to determine whether volatile operation can be retained under deformation. Devices fabricated with pristine semiconducting films maintained stable operation only up to 20% strain, with device failure occurring at 30% strain (Figure S5). In contrast, OECTs incorporating the SEBS‐blended channel retained gate‐controlled modulation at 30% strain, demonstrating the effectiveness of the elastomeric matrix in maintaining electrical continuity under deformation (Figure 3a). The dynamic electrical performance of the stretchable devices was further examined. Frequency‐dependent transconductance measurements revealed a gradual decrease in response beyond 5 Hz, consistent with the relatively large channel capacitance that limits high‐frequency operation (Figure 3b). Nevertheless, pulse‐resolved sinusoidal outputs remained distinguishable under tensile strains up to 30% (Figure 3c). Transient switching characteristics, obtained by applying a −0.6 V gate pulse, showed reversible gate‐controlled modulation at all tested strain levels, although both the modulation amplitude and response speed changed with increasing strain (Figure 3d). The rising and falling times increased by factors of 2.14 and 2.86, respectively, at 30% strain, compared with the unstrained condition (Figure S12). Part of the attenuation in drain current and transconductance is intrinsically expected from the strain‐induced reduction in the channel geometrical factor, which scales approximately as λ^−^^2^ under affine and incompressible deformation. However, the experimentally measured attenuation exceeded this geometrical prediction (Figure S13 and Note S1). Together with the strain‐dependent increases in gate impedance and electrode resistance, these results indicate combined contributions from channel geometry, gate/electrolyte coupling, and electronic series or contact resistance, rather than a single mechanism such as irreversible channel fracture or permanent ion trapping. The closely overlapping transfer characteristics of a separately tested P(g2T2‐T)/SEBS‐based OECT under strains up to 50% further indicate that deterioration of the intrinsic bulk‐channel properties is not the sole origin of the performance reduction (Figure S6). The larger strain dependence compared with nanowire‐ and Ag/AgCl‐paste‐based OECTs is consistent with the crack‐mediated thin‐film electrodes and multiple interfaces of the fully integrated dual‐gate architecture, rather than with the intrinsic suitability of Ag/AgCl as a stretchable gate material (Table S1). The applicability of the volatile OECT platform for bioelectronic sensing was further evaluated by applying an electrocardiogram (ECG)‐like signal (1 Hz, 10 mV peak‐to‐peak; Figure S14) to the gate electrode (Figure 3e). The device reproduced the characteristic temporal features of the input waveform at tensile strains up to 30%, although the output‐current amplitude decreased with increasing strain. Thus, the SEBS‐based architecture enables functional waveform transduction under deformation but does not provide strain‐invariant electrical performance. The present platform is therefore more appropriately described as mechanically stretchable and functionally tolerant to strain, with measurable strain‐dependent changes in current magnitude and response dynamics. Although the response speed is limited by the channel capacitance, it may be further improved through geometric optimization of the channel dimensions. These characteristics highlight the potential of the developed system for integration into soft bioelectronic platforms, including wearable sensing and closed‐loop therapeutic systems.

While the volatile operation enabled by the Ag/AgCl gate is well‐suited for transient signal detection, it does not provide the capability to store or accumulate information. To address this limitation, we next exploit the non‐volatile operation of the same device by using the Au gate, thereby enabling bifunctional operation within a single stretchable dual‐gate OECT platform. Under Au gate operation a pronounced hysteresis remained observable under tensile strains up to 30%, indicating that non‐volatile conductance modulation was retained during mechanical deformation (Figure 3f). However, the absolute current level and hysteresis characteristics changed with increasing strain. These results therefore indicate preservation of non‐volatile functionality under deformation rather than strain‐invariant memory characteristics. Because the conductance state of the channel directly corresponds to the synaptic weight in biological systems, such hysteretic behavior suggests that the device can store electrical stimuli and therefore emulate synaptic memory.

Dynamic synaptic responses were evaluated by applying presynaptic voltage pulses to the gate electrode while monitoring the postsynaptic current (PSC) measured between the source and drain electrodes. Upon application of a gate pulse, ion injection and extraction processes occur within the channel, leading to modulation of the channel conductance and corresponding changes in the drain current. When negative presynaptic pulses are applied to the gate electrode (amplitude = −0.05, −0.1, and −0.15 V; pulse width = 25 ms; base voltage = 0.7 V, drain voltage = −0.6 V), excitatory postsynaptic currents (EPSC) are induced (Figure 3g), where ion injection into the channel leads to an increase in conductance. In contrast, positive presynaptic pulses (amplitude = 0.05, 0.1, and 0.15 V; pulse width = 25 ms; base voltage = 0.7 V, drain voltage = −0.6 V) induce inhibitory postsynaptic currents (IPSC) (Figure S15), where ion extraction from the channel leads to a reduction in conductance. The magnitude of the EPSC increases with pulse amplitude, indicating that a larger number of ions are injected into the channel at higher gate biases, resulting in a higher degree of channel doping. Notably, the current does not fully return to its initial baseline after the pulse stimulation, which can be reflected to the partial retention of ions within the P(g2T2‐T):SEBS channel. This residual ionic state contributes to conductance accumulation, forming the basis of synaptic plasticity in the device.

Short‐term synaptic plasticity was investigated through paired‐pulse measurements by applying two consecutive presynaptic pulses (−0.1 V for EPSC and 0.1 V for IPSC) with different time intervals (*Δ* *t*), using a fixed pulse width of 50 ms and a drain voltage of −0.6 V. The Au gate OECT exhibits paired‐pulse facilitation (PPF), where the second EPSC peak (*A*_2_) is enhanced relative to the first (*A*_1_) at short *Δ* *t* due to residual ionic states in the channel, and the PPF index (*A*_2_/*A*_1_) decreases with increasing *Δ* *t* (Figure 3h). The decay behavior can be fitted using a double exponential function (Equation 1), yielding characteristic time constants associated with fast and slow ionic relaxation:

(1) $$PPF=C_{0}+C_{1}e^{-\frac{\Delta t}{\tau_{1}}}+C_{2}e^{-\frac{\Delta t}{\tau_{2}}}$$

Here, *C*_1_ and *C*_2_ represent the initial facilitation magnitudes for fast and slow components, while *τ*_1_ and *τ*_2_ denote the respective relaxation time constants. The fitted curve shows good agreement with the experimental data (*R* ^2^ = 0.8036), with *τ*_1_ = 6.32 ms and *τ*_2_ = 305.16 ms, indicating the coexistence of rapid and slow ionic relaxation processes. Under opposite polarity, paired‐pulse depression (PPD) is also observed, where the second response is suppressed due to ion depletion induced by the first pulse (Figure S16). The corresponding PPD can also be fitted using double exponential function (*R* ^2^ = 0.8338), with *τ*_1_ = 10.83 ms and *τ*_2_ = 118.13 ms. These results confirm that the device reproduces bidirectional short‐term synaptic plasticity governed by ion dynamics. The inset of Figure 3h and Figure S16 show representative PSC at *Δ* *t* = 75 ms, clearly illustrating the bidirectional facilitation and depression, respectively.

Long‐term synaptic plasticity was further evaluated by applying 100 presynaptic pulse trains to the gate electrode to induce long‐term potentiation (LTP) and long‐term depression (LTD). The conductance gradually increases and decreases under sequential potentiation and depression pulses, demonstrating analog weight modulation and enabling the emulation of long‐term memory behavior. Such characteristics are critical for neuromorphic systems, as parameters extracted from LTP/LTD responses, such as asymmetricity (*AS = NL_P_ − NL_D_*, where NL denotes nonlinearity) [55] and dynamic range (*G*_max_/*G*_min_), directly affect the learning accuracy of artificial neural networks. A detailed calculation of AS and dynamic range is provided in the Experimental Section/Methods. To optimize synaptic performance, the PSC were systematically measured by varying the potentiation pulse amplitude, depression pulse amplitude, and pulse frequency (Figure S17a,c,e and Table S3). The resulting LTP/LTD characteristics were analyzed to extract AS and dynamic range under each condition (Figure S17b,d,f and Table S3). As the pulse parameters changed, both AS and dynamic range exhibited strong dependence on the stimulation conditions, highlighting the critical role of pulse optimization for achieving balanced synaptic weight updates.

Based on this analysis, the optimal pulse condition was determined as −0.05 V for potentiation and 0.1 V for depression with a pulse frequency of 20 Hz, providing a favorable trade‐off between asymmetricity of 7.76 and dynamic range of 2.87. Using these optimized conditions, LTP/LTD characteristics were obtained by applying 100 consecutive potentiation and depression pulses, as shown in Figure 3i. Importantly, these characteristics were preserved under tensile strain up to 30%. The corresponding evolution of asymmetricity and dynamic range under strain is summarized in Figure S18 and Table S4.

We note that the 100 applied pulses correspond to the number of programming pulses, not to the number of physically distinguishable conductance states. Following the established definition of effective conductance states [56], in which a state is counted as usable only when its per‐pulse conductance increment (*ΔG*) exceeds a threshold (threshold*_ΔG_* = 0.3% of *G_max_ − G_min_*), the number of effective conductance states decreases from 96 at 0% strain to 91, 74, and 66 at 10%, 20%, and 30% strain, respectively (Figure S19, and S20). This reduction reflects the increased weight‐update nonlinearity and the narrowing of the conductance window (dynamic range decreasing from ∼2.87) under strain. These effective conductance states, rather than the nominal pulse count, were used for the neural network simulation described below.

To validate the impact of synaptic characteristics on neuromorphic performance, the artificial neural network (ANN) was trained for Modified National Institute of Standards and Technology (MNIST) handwritten digit dataset recognition based on multilayer perceptron (MLP) model (Figure 3j). The network was trained using conductance updates derived from the optimized LTP/LTD characteristics (Figure 3i), and the resulting training accuracy under strain is shown in Figure 3k. The recognition accuracy decreases gradually with increasing strain, from ∼91.2% at 0% to ∼90.8% (10%), ∼89.1% (20%), and ∼85.2% (30%) (averaged over the last 10 training epochs; inset of Figure 3k), reflecting the strain‐dependent reduction in the number of effective conductance states rather than strain‐invariant performance. Notably, the network still maintains a recognition accuracy above 85% even at 30% strain, indicating that the synaptic weight update behavior remains functional under mechanical deformation. A comparison of classification accuracy under different pulse conditions is provided in Figure S21, highlighting the importance of pulse optimization for achieving improved neuromorphic performance. Furthermore, a comprehensive comparison with previously reported synaptic devices is summarized in Table S5, including AS, dynamic range, number of conductance states, and recognition accuracy [57]. This comparison demonstrates that our Au‐gate stretchable OECT achieves a competitive balance between weight update, dynamic range, and classification accuracy, while additionally offering intrinsic stretchability.

### 2.4 Pavlovian Associative Learning Using Intrinsically Stretchable Bifunctional Dual‐Gate OECT

**FIGURE 4:**
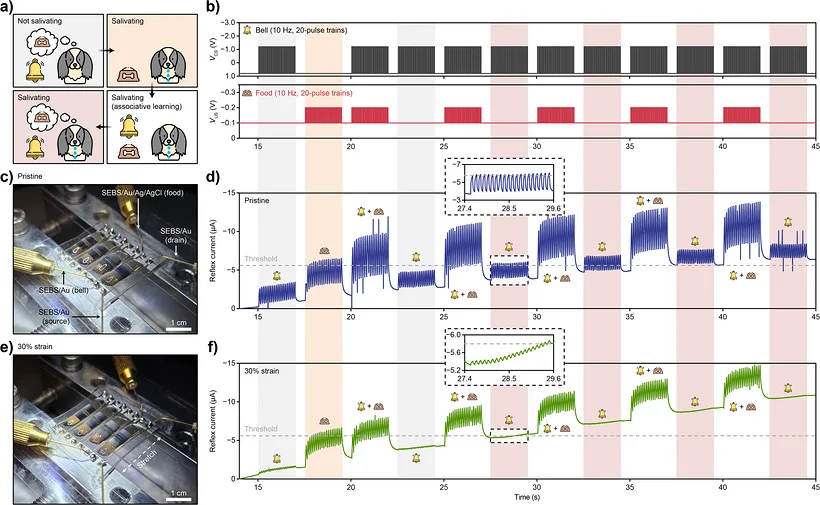
Pavlovian associative learning emulated by intrinsically stretchable bifunctional dual‐gate OECTs. (a) Schematic of Pavlov's dog experiment, in which an initially neutral bell stimulus does not induce salivation, whereas a food stimulus evokes salivation. After repeated paired stimulation of bell and food, the bell alone becomes a conditioned stimulus capable of inducing salivation through associative learning. The icons of the dog, bell, and food were obtained from free icon resources. Designed by Magnific (www.magnific.com) (b) Input voltage pulse sequences applied to the dual‐gate OECT. Bell stimuli were applied to the SEBS/Au gate as non‐volatile‐mode inputs, and food stimuli were applied to the SEBS/Au/Ag/AgCl gate as volatile‐mode inputs. Both stimuli were delivered as 10 Hz, 20‐pulse trains. *V*_CS_, voltage input for the conditioned stimulus; *V*_US_, voltage input for the unconditioned stimulus. (c) Photograph of the pristine dual‐gate OECTs during the Pavlovian learning experiment, showing the SEBS/Au gate for bell inputs, the SEBS/Au/Ag/AgCl gate for food inputs, and the SEBS/Au source/drain electrodes. (d) Reflex current response of the pristine dual‐gate OECTs during sequential bell, food, and paired bell‐food stimulation. The initial food stimulus evokes an immediate volatile reflex response, defining the threshold current level for salivation‐like output. Repeated paired stimulation of bell and food progressively strengthens the bell‐associated non‐volatile response, eventually enabling the bell‐only stimulus to induce a reflex current exceeding the threshold. The inset magnifies the moment at which the bell‐evoked response first surpasses the threshold defined by the initial food‐induced current level. (e) Photograph of the dual‐gate OECTs under 30% tensile strain during the Pavlovian learning experiment, demonstrating mechanically stretchable operation. (f) Reflex current response of the dual‐gate OECTs under 30% strain. The intrinsically stretchable dual‐gate OECT preserves associative‐learning behavior under mechanical deformation, where repeated paired bell‐food stimulation enables the bell‐only stimulus to induce a conditioned reflex current above the threshold. The inset magnifies the threshold‐crossing region under 30% strain.

Building upon the demonstrated bifunctional operation of the dual‐gate OECT, we further explored its capability to integrate volatile sensing and non‐volatile memory within a single device by emulating Pavlovian associative learning [34, 44, 58]. In the classical conditioning paradigm (Figure 4a), a bell stimulus initially does not induce a salivation response, whereas a food stimulus evokes an immediate reflex. Through repeated paired presentation of the bell and food, the response becomes conditioned through the association between the two stimuli, eventually enabling the bell alone to trigger the conditioned response. In our platform, the SEBS/Au/Ag/AgCl gate operates as a volatile sensing pathway corresponding to the unconditioned stimulus (US, food), while the SEBS/Au gate functions as a non‐volatile memory pathway corresponding to the conditioned stimulus (CS, bell) (Figure 4b,c). Voltage pulse trains were independently applied to each gate, where the US input was delivered as −0.1 V amplitude pulses with a −0.1 V base at 10 Hz for 20 pulses, and the CS input was applied as −2 V amplitude pulses with a 0.8 V base at 10 Hz for 20 pulses. The resulting drain current was monitored as the output signal.

The changes of the device response during the associative learning process are shown in Figure 4d. When the CS was initially applied alone, the device exhibited only a weak response that remained below the predefined threshold. In contrast, the US induced an immediate transient current exceeding the threshold due to the volatile operation of the Ag/AgCl gate. During repeated paired stimulation of the CS and US, the response to the CS progressively increased, reflecting the accumulation of non‐volatile conductance states induced by the Au gate. After the first paired stimulation cycle, the CS‐only response increased but still remained below the threshold. Notably, after the second cycle, the CS‐only stimulus became sufficient to exceed the threshold, indicating the formation of a conditioned response. The inset in Figure 4d highlights this first threshold‐crossing event of the CS‐induced signal.

The same experimental sequence was applied under a tensile strain of 30% (Figures 4e,f). Figure 4e shows a photograph of the bifunctional dual‐gate OECT under applied strain during the associative learning experiments, confirming operation under deformation. Although the absolute current level was reduced under mechanical deformation, the sequence‐dependent threshold‐crossing outcome was retained. The CS‐only response remained below the predefined threshold after the first paired cycle but exceeded it after the second cycle (Figure 4f). The inset of Figure 4f further confirms the preserved threshold‐crossing behavior under strain. These results demonstrate that the intrinsically stretchable dual‐gate OECT enables bifunctional operation by co‐localizing volatile sensing and non‐volatile memory to realize adaptive neuromorphic functionalities, while maintaining operation under mechanical deformation, providing a viable platform for next‐generation soft brain‐inspired OECT platforms.

## 3 Conclusion

We have reported an intrinsically stretchable OECT that integrates sensing, memory and neuromorphic functionality in a single platform through gate metal‐driven bifunctionality, assembled from an ionically conductive hydrogel and an elastic semiconductor (PSES) based on percolative phase separation. With selectively addressed gate electrodes, PSES enables reversible responses for transient signal detection, yet on demand modulates channel conductance for synapse‐like memory behavior and is directly utilized to implement artificial neural network (ANN), thereby bypassing the limitations of previous approaches, in which bifunctional operation was confined to rigid devices and stretchable OECTs were limited to a single operation mode with only channel‐level stretchability. Furthermore, the bifunctional gate modulation enables associative learning, in which repeated paired stimuli progressively strengthen the device response, and the intrinsically stretchable hydrogel/semiconductor bilayer membrane maintains operation under mechanical deformation despite strain‐dependent reductions of performance, underscoring their potential for brain‐inspired neuromorphic computing and closed‐loop neuroprosthetics. To improve the computing complexity, a scalable method for stretchable OECT arrays and circuits will be needed.

## 4 Experimental Section/Methods

### 4.1 Materials

The chemicals used in the study, including Poly[3,3*′*‐bis[2‐[2‐(2‐methoxyethoxy)ethoxy]ethoxy]‐2,2*′*:5*′*,2*′′*‐terthiophene‐5,5*′′*‐diyl], was purchased from Ossila. Alginic acid sodium salt (from brown algae, medium viscosity) was purchased from Sigma‐Aldrich. SEBS was obtained from Asahi Kasei.

### 4.2 TOF‐SIMS Analysis of the P(g2T2‐T)/SEBS Films

Time‐of‐flight secondary‐ion mass spectrometry (TOF‐SIMS) experiments were conducted using a TOF‐SIMS‐5 instrument (ION‐TOF GmbH) equipped with a Bi3+ liquid metal ion gun, an oxygen sputtering gun, and a low energy electron flood gun for charge compensation. Cs+ with an energy of 1 keV energy and a 40 nA current was used as the sputter source. The typical sputter area was 150 µm by 150 µm. The spatial resolution was under 300 nm. Depth profiles of S− and C− secondary ions were collected and normalized as a function of sputtering time.

### 4.3 Nanoindentation Measurements

Nanoindentation tests were performed using the NanoTest Vantage Platform (Micro Materials) to evaluate the mechanical characteristics of the P(g2T2‐T)/SEBS films. The samples were then cut into rectangular shapes of 10‐mm width and length for measuring the hardness, reduced modulus and elastic recovery ratio with a diamond Berkovich indenter tip (sharpness of 56.36 nm and an effective radius of 68.67 ± 1.48 nm).

### 4.4 Semiconducting Elastomer and Ionically Conductive Hydrogel Film Fabrication

The ionically conductive hydrogel solution (30 mg mL^−1^), prepared by dissolving it in deionized (DI) water at 25°C for 6 h, was spin‐coated at 2 000 rpm for 2 min onto Si substrates that had been sequentially cleaned with acetone, isopropyl alcohol, and DI water. The semiconductor solution (7 mg mL^−1^) was prepared by dissolving it in chloroform at 25°C for 4 h. To fabricate the bilayer film, the semiconductor solution was spin‐coated at 700 rpm for 1 min on the ionically conductive hydrogel film on the Si substrate. Next, the entire film was exfoliated from the Si substrate using a supporting sticker frame to maintain its structural integrity. Subsequently, the frame was removed by trimming the edges of the sticker, which resulted in a free‐standing bilayer film.

### 4.5 Electrochemical Characterization

Electrochemical properties of the semiconducting films were investigated using cyclic voltammetry (CV), electrochemical impedance spectroscopy (EIS), and chronoamperometry. All measurements were performed using a potentiostat (ZIVE SP1, ZIVELAB) in a three‐electrode configuration with the polymer film as the working electrode, Ag/AgCl reference electrode, and platinum mesh counter electrode. The electrolyte consisted of 1× PBS (pH 7.4). CV measurements were conducted over a potential window of 0.4 to ‐0.8 V at scan rates ranging from 5 to 150 mV s^−^^1^. The relationship between peak current (*i*) and scan rate (*v*) was analyzed according to a power‐law dependence *i = a·v^b^*, where the b‐value was determined from the slope of the log(*i*) vs. log(*v*) plot. EIS measurements were performed over a frequency range of 10 mHz to 1 kHz with an AC perturbation amplitude of 10 mV at a DC bias of 0 V. The impedance spectra were fitted using an equivalent circuit model consisting of *R*_s_, *R*_ct_, *W*, *C*_dl_ using ZIVE ZMAN 2.5 software. Parameters associated with charge storage and ion transport, including capacitance and diffusion‐related elements, were extracted from the fitting results. Chronoamperometric measurements were carried out by applying a voltage pulse of −0.4 V and recording the resulting current response. The transient behavior was analyzed by fitting the current decay to an exponential function using OriginPro software to extract characteristic time constants associated with ion transport and relaxation dynamics.

### 4.6 Strain‐Dependent Electrochemical Impedance Characterization of Gate Electrodes

SEBS/Au, SEBS/Au/Ag/AgCl, and SEBS/Ag/AgCl electrodes were mounted on a uniaxial stretching stage and elongated to tensile strains of 0, 10%, 20%, and 30%. Electrochemical impedance spectroscopy was performed at each strain condition using a potentiostat (ZIVE SP1, ZIVELAB) in three‐electrode configuration. The measurements were conducted in 1× PBS (pH 7.4) using an AC perturbation amplitude of 10 mV at a DC bias of 0 V over a frequency range of 0.1 Hz to 10 kHz. The exposed electrode area was maintained at 12 mm^2^ for SEBS/Au and SEBS/Ag/AgCl, 75 mm^2^ for SEBS/Au/Ag/AgCl using Foam Tape Clear Gel (Alpha Co. Seoul, Republic of Korea).

### 4.7 OECT Device Fabrication for Electrical Characterization

The glass substrate was cleaned sequentially with acetone, isopropyl alcohol and DI water. The source/drain Cr/Au electrodes (10/50 nm thick) were patterned through thermal evaporation using a mask aligner (MA6 Gen4, SUSS MicroTec SE, NFEC‐2025‐08‐307674, Germany), operated at the i‐line (365 nm).

### 4.8 Evaluation of Volatile Operation and Device Performance

The electrical characteristics of the OECT devices were evaluated using a semiconductor parameter analyzer and/or source‐measure unit (B1500A, Agilent) equipped with a probe station (MSTECH). Transfer characteristics were measured with the B1500A by sweeping the gate voltage from +0.4 to −0.8 V and back (double sweep) in steps of 12 mV at a fixed drain voltage of −0.6 V, with the hold and delay times set to 0 s and using the high‐resolution ADC. The measurement was performed in stepped‐sweep mode; we therefore specify the voltage step and timing conditions rather than a nominal sweep rate. To investigate volatile operation, gate‐dependent switching behavior was examined using Ag/AgCl‐gate electrodes under identical measurement conditions. Transient switching characteristics were measured by applying a gate voltage pulse of −0.6 V using a function generator (Analog discovery studio, Digilent) while monitoring the drain current response with (B1500A, Agilent). Rising and falling times were extracted as the time required for the current to reach 10%–90% of steady‐state value. Frequency‐dependent transconductance measurements were performed by applying sinusoidal gate voltages with frequencies ranging from 0.1 Hz to 1k Hz, and the corresponding drain current response was recorded to evaluate dynamic device behavior. Mechanical stability under deformation was assessed by applying uniaxial strain using a manual stretcher. To evaluate the capability of the device for biosignal sensing, an ECG‐like signal (1 Hz, 10 mV peak‐to‐peak) was generated using a function generator (Analog discovery studio, Digilent) and applied to the gate electrode. For all measurements under strain, uniaxial tensile strain was applied parallel to the channel length direction using a custom‐made manual stretcher.

### 4.9 Evaluation of Non‐Volatile Operation and Device Performance

The electrical characteristics of the non‐volatile OECT devices were evaluated using a semiconductor parameter analyzer (B1500A, Agilent) equipped with a probe station (MSTECH). Transfer characteristics were measured under Au‐gate operation to analyze the hysteretic behavior associated with non‐volatile conductance modulation, by sweeping the gate voltage from +1.5 to −2 V and back (double sweep) in steps of 35 mV at a fixed drain voltage of −0.6 V, with a hold and delay times set to 0 s and using the high‐resolution ADC. Synaptic characteristics were investigated by applying gate voltage pulses using the semiconductor pulse generator unit (SPGU) integrated in the B1500A system, while monitoring the drain current as the postsynaptic response. Excitatory and inhibitory postsynaptic current (EPSC/IPSC) responses were obtained by applying voltage pulses with controlled amplitude, polarity, and pulse width to the gate electrode. Short‐term synaptic plasticity was evaluated through paired‐pulse measurements, where two consecutive gate pulses were applied with varying inter‐spike intervals to extract PPF and PPD. The PPF and PPD response was further analyzed by fitting the decay of the facilitation index to a double‐exponential function using OriginPro software, allowing extraction of characteristic time constants associated with fast and slow ion relaxation processes. Long‐term synaptic plasticity was characterized by applying repeated 100 pulse trains to induce LTP and LTD, enabling gradual conductance modulation. For all measurements under strain, uniaxial tensile strain was applied parallel to the channel length direction using a custom‐made manual stretcher.

### 4.10 Weight Update for Artificial Synaptic Device

During synaptic weight updating, the difference between the target and output values (*δ* = target value—output value) is used to determine the direction of conductance modulation. The synaptic weight is defined as *W*  = *G* ^+^  − *G* ^−^_,_ where *G* ^+^ and *G* ^−^ represent the conductance states of the positive and negative channels, respectively. For *δ* > 0, the synaptic weight is potentiated by increasing *G* ^+^ and decreasing *G* ^−^ simultaneously, whereas for *δ* < 0, the weight is depressed by decreasing *G* ^+^ and increasing *G* ^−^ simultaneously. The conduction update of each synapse is calculated by the following equations:

(2) $$G_{n+1}=G_{n}+\Delta \;G_{P}=G_{n}+\alpha_{P}e^{-\beta_{P}\frac{G_{n}-G_{min}}{G_{max}-G_{min}}}\;\left(\Delta G>0\right)$$

(3) $$\;G_{n+1}=G_{n}+\Delta \;G_{D}=G_{n}\;-\alpha_{D}e^{-\beta_{D}\frac{G_{max}-G_{n}}{G_{max}-G_{min}}}\;\left(\Delta G<0\right)$$

where *G_n_* and *G*_*n* + 1_ denote the conductance values after *n* ^th^ and (*n*+1)^th^ pulses, respectively. The parameters *α* and *β* represent the conductance update magnitude and nonlinearity, respectively.

### 4.11 Pavlovian Associative Learning Experiment Using Dual‐Gate OECT

Pavlovian associative learning was implemented using the intrinsically stretchable dual‐gate OECT by independently applying voltage pulse trains to the two gate electrodes corresponding to conditioned and unconditioned stimuli. The SEBS/Au gate was used to deliver the conditioned stimulus (CS, bell), while the SEBS/Au/Ag/AgCl gate was used to deliver the unconditioned stimulus (US, food). Voltage pulses were applied using two SPGUs integrated in a semiconductor parameter analyzer (B1500A, Agilent), enabling independent control of the two gate inputs. The CS and US inputs were applied as pulse trains with defined amplitude, base voltage, frequency, and number of pulses. The resulting drain current was simultaneously recorded using a SMU to monitor the device response during sequential and paired stimulation. Associative learning behavior was evaluated by comparing the drain current responses under CS‐only, US‐only, and paired CS‐US stimulation conditions, as well as by tracking the evolution of the CS‐induced response during repeated pairing cycles in real time.

## Author Contributions

D. Son conceived of and supervised the research; J.Y., J.J. and H.J. designed and fabricated the electronic devices and performed their characterization; J.Y. designed the Pavlovian associative learning set‐ups, with assistance from D. Seong; H.J. performed the static characteristics measurements, with assistance from Y.J.; J.J. performed the transient characteristics measurements, with assistance from D.K.; J.Y. performed the ANN simulation using conductance modulation measurements, with assistance from S.M.W.; J.Y., J.J., H.J. and D. Son wrote the manuscript and prepared the figures. J.H.K. reviewed and refined the revised manuscript. All authors commented on the paper.

## Conflicts of Interest

The authors declare no conflicts of interest.

## Supporting information




**Supporting File**: advs77505‐sup‐0001‐SuppMat.docx.

## Data Availability

The data that support the findings of this study are available from the corresponding author upon reasonable request.
