# Supplementary material for: Gate Metal‐Driven Sensing and Memory Bifunctionality in Intrinsically Stretchable Organic Electrochemical Transistors for Soft Neuromorphic Systems
Source: Adv Sci (Weinh). 2026 Aug 30:e77505. Online ahead of print. doi: 10.1002/advs.77505 (PMC13526708; doi:10.1002/advs.77505)
Supplement: Supplementary file 1 [file ADVS-9999-e77505-s001.docx]

Gate Metal-Driven Sensing and Memory Bifunctionality in Intrinsically Stretchable Organic Electrochemical Transistors for Soft Neuromorphic Systems

Jiyong Yoon, Jaepyo Jang, Hyunjin Jung, Duhwan Seong, Daeun Kim, Yewon Jeon, Ja Hoon Koo, Sang Min Won and Donghee Son*

J. Yoon, J. Jang, H. Jung, D. Seong, S. M. Won

Department of Electrical and Computer Engineering

Sungkyunkwan University

Suwon, 16419, Republic of Korea

J. Yoon, J. Jang, H. Jung, D. Seong, D. Son

Center for Neuroscience Imaging Research

Institute for Basic Science

Suwon, 16419, Republic of Korea

E-mail: daniel3600@yonsei.ac.kr

D. Kim, D. Son

Department of Chemical and Biomolecular Engineering

Yonsei University

Seoul, 03722, Republic of Korea

Y. Jeon

Department of Chemistry

Ajou University

Suwon, 16499, Republic of Korea

J. H. Koo

Department of Semiconductor Systems Engineering

Sejong University

Seoul, 05006, Republic of Korea

Jiyong Yoon, Jaepyo Jang and Hyunjin Jung contributed equally to this work.

Keywords: Stretchable electronics, Organic electrochemical transistors, Bifunctional operation, Associative learning

**This PDF file includes:**

Supplementary Note. S1

Supplementary Figure. S1–21

Supplementary Table. S1–5

**Supplementary Note**

**Supplementary Note S1. Geometrical contribution to the strain-dependent attenuation of OECT performance**

To estimate the intrinsic geometrical contribution to the strain-dependent changes in drain current and transconductance, we considered the deformation of the OECT channel under uniaxial tensile strain applied parallel to the source–drain direction. The engineering strain is denoted by ε, and the corresponding stretch ratio is defined as $\lambda=1+\varepsilon$. The initial channel length, width, and thickness are denoted by $L_{0}$, $W_{0}$, and $d_{0}$, respectively. Under tensile deformation along the channel-length direction, the channel length increases according to $L_{\varepsilon}=\lambda L_{0}$. The P(g2T2-T) channel is approximated as an incompressible elastomeric composite undergoing homogeneous affine deformation. Under this assumption, the channel volume is conserved $L_{0}W_{0}d_{0}.$ Assuming equivalent transverse contraction along the width and thickness directions, the two transverse stretch ratios are both $\lambda^{-1/2}$. Therefore$W_{\varepsilon}=W_{0}\lambda^{-1/2}$and $d_{\varepsilon}=d_{0}\lambda^{-1/2}$. For an OECT with unchanged charge-carrier mobility, volumetric capacitance, threshold voltage, and electrochemical gating efficiency, both the drain current and transconductance scale approximately with the channel geometrical factor $\frac{Wd}{L}$. The strain-dependent geometrical factor is therefore $\frac{W_{0}d_{0}}{L_{0}}\lambda^{-2}$.

**Supplementary Figures**

**Figure S1. Optical microscopy images of the pure P(g2T2-T) and the phase-separated morphology of the elastic semiconductor (PSES).**

**Figure S2. Optical microscopy images of the P(g2T2-T)/SEBS films with increasing P(g2T2-T) volume ratios under tensile strain.**

**Figure S3. TOF-SIMS analysis of the P(g2T2-T)/SEBS films with increasing P(g2T2-T) volume ratios.**

**Figure S4. Nanoindentation elastic modulus of the P(g2T2-T)/SEBS films with increasing P(g2T2-T) volume ratios, showing enhanced softness with increasing polymer fraction.** Data are expressed as the mean ± s.d. (*n* = 4 for independent samples). *P* = 0.0052 for the comparison between 70 vol% and 100 vol% P(g2T2-T).

**Figure S5. Transfer characteristics of the P(g2T2-T)/hydrogel bilayer membrane-based dual-gate OECTs operated in volatile mode using the SEBS/Au/Ag/AgCl gate (a) and non-volatile mode using the SEBS/Au gate (b) under tensile strains of 0, 10, 20 and 30%.** The device exhibited strain-induced degradation and failed to maintain stable OECT operation at 30% strain.

**Figure S6. Transfer characteristic changes of the P(g2T2-T)/SEBS-based OECT under stretching parallel to the charge transport direction with strain from 0% to 30% and 50%, with a channel geometry of *W/L* = 3,000/200 μm, measured at *V*_DS_ = −0.6 V.**

**Figure S7. Fabrication process of the elastic semiconductor channel and intrinsically stretchable dual-gate OECTs. a)** Schematic of the fabrication process for the semiconductor channel. An ion-reserving hydrogel layer is first coated on the substrate, followed by orthogonal layered assembly (OLA) of the semiconductor interface to form a hydrogel/semiconductor bilayer membrane. The channel region is then defined by selective isolation using CHCl₃, and the entire film is exfoliated from the substrate to obtain a freestanding semiconductor channel. **b)** Schematic of the fabrication process for intrinsically stretchable dual-gate OECTs. A SEBS layer is first drop-cast to form an elastic substrate, followed by patterning of Au/Ag electrodes. The Ag layer is chemically chlorinated by immersion in 0.1 M FeCl₃ solution to form Ag/AgCl gate electrodes. The freestanding semiconductor channel is then transferred and bonded onto the patterned SEBS/Au/Ag/AgCl platform, resulting in a fully stretchable dual-gate OECT architecture comprising source/drain electrodes, an Au gate, and an Ag/AgCl gate.

**Figure S8. Photographs showing the chemical chlorination of Ag deposited on SEBS/Au electrodes in FeCl₃ solution to form AgCl, according to the reaction Ag(s) + FeCl₃(aq) → AgCl(s) + FeCl₂(aq).**

**Figure S9. Areal impedance spectra of SEBS/Ag/AgCl electrode under tensile strains of 0, 10, 20 and 30%.**

**Figure S10. Electrochemical and dynamic characterization of the pure P(g2T2-T). a)** CV of the pure P(g2T2-T) measured at different scan rates from 5 to 150 mV s⁻^1^, showing reversible electrochemical doping and dedoping behavior. **b)** Logarithmic plot of the peak current as a function of voltage sweep rate. The power-law fitting gives a b-value, indicating predominantly surface-confined/capacitive charge-storage behavior with partial diffusion contribution in the pure P(g2T2-T). **c)** EIS impedance spectra of the pure P(g2T2-T), showing the frequency-dependent impedance modulus and phase response. **d)** Nyquist plots for the pure P(g2T2-T). **e)** Transient current response of the pure P(g2T2-T) after voltage biasing, showing rapid current relaxation with a time constant of *τ*, indicating ion–electron coupling dynamics in the pure P(g2T2-T) channel.

**Figure S11. Nyquist plots for the PSES.**

**Figure S12. Quantified rising and falling times of the volatile-mode OECT extracted from the dynamic switching responses under tensile strains of 0, 10, 20 and 30%.** Both the rising and falling times gradually increase with increasing strain, indicating a moderate strain-dependent slowing of the transient response while maintaining stable volatile operation.

 **Figure S13. Geometrical and electrochemical contributions to the strain-dependent performance attenuation of the Ag/AgCl-gated stretchable OECT. a)** Schematic illustrations of the OECT channel in the unstrained and tensile-strained states. When uniaxial tensile strain is applied along the source–drain direction, the channel length ($L$) increases, whereas the channel width ($W$) and thickness ($d$) decrease through transverse contraction, resulting in a reduction in the geometrical factor $Wd/L$. **b)** Comparison of the normalized geometrical factor ($Wd/L$), Ag/AgCl gate-electrode impedance ($Z_{G}/Z_{G0}$), drain current ($I_{D}/I_{D0}$), and transconductance ($g_{m}/g_{m0}$) at tensile strains of 0, 10, 20, and 30%. The geometrical factor was calculated assuming approximately affine and incompressible deformation, and all parameters were normalized to their respective values at 0% strain. $Z_{G}$represents the areal impedance of the SEBS/Au/Ag/AgCl gate electrode measured at 5 Hz. The decrease in $I_{D}$and $g_{m}$is greater than that predicted solely from the change in $Wd/L$, while the gate impedance increases upon deformation, suggesting that both device-level geometrical deformation and strain-dependent gate/electrolyte coupling contribute to the attenuation of the volatile-mode OECT response.

**Figure S14. Representative ECG waveform applied as a physiological input to the intrinsically stretchable OECT operated in volatile mode.** The ECG showed a characteristic cardiac waveform with a dominant frequency of 1 Hz, corresponding to 60 bpm, which is comparable to a normal resting human heart rate.

**Figure S15. Inhibitory post-synaptic current (IPSC) responses of the non-volatile-mode OECT triggered by single voltage pulse with different amplitude.** The single pulse stimulation was applied to the SEBS/Au gate, and the resulting IPSC was recorded from the channel.

**Figure S16. Paired-pulse depression (PPD) index (*A*_2_/*A*_1_) as a function of the inter-spike interval (*Δt*).** The PPD behavior is fitted, showing short- and long-term relaxation components with time constants of τ_1_ and τ_2_. The inset defines *A*_1_ and *A*_2_ from two sequential IPSC peaks at *Δt* = 75 ms.

**Figure S17. Pulse-dependent synaptic plasticity characteristics of the non-volatile-mode OECT. a,b)** Conductance modulation of the non-volatile-mode OECT under repeated potentiation/depression pulse trains with different LTP pulse amplitudes while the LTD pulse amplitude and pulse frequency are fixed (a) and the corresponding asymmetry and dynamic range values (b). **c,d)** Conductance modulation of the non-volatile-mode OECT under repeated potentiation/depression pulse trains with different LTD pulse amplitudes while the LTP pulse amplitude and pulse frequency are fixed (c) and the corresponding asymmetry and dynamic range values (d). **e,f)** Conductance modulation of the non-volatile-mode OECT under repeated potentiation/depression pulse trains with different pulse frequency while both LTP and LTD pulse amplitudes are fixed (e) and the corresponding asymmetry and dynamic range values (f).

**Figure S18. The asymmetricity and dynamic range extracted from the potentiation/depression characteristics measured at 0, 10, 20 and 30% strain.** The gradual increase in asymmetry and decrease in dynamic range with increasing strain indicate strain-dependent changes in synaptic weight update linearity and conductance modulation window.

**Figure S19. Extraction of usable conductance states from the LTP characteristics under strain.** Per-pulse conductance increment (*ΔG*) along the LTP trajectory as a function of pulse number under (a) 0%, (b) 10%, (c) 20% and (d) 30% strain. The red dashed line in each panel denotes threshold*_ΔG_*, set to 0.3% of the available conductance window (*G_max_ − G_min_*) at the corresponding strain. Pulses whose ΔG falls below this threshold lie in the saturated, strongly nonlinear region of the LTP curve and are excluded from the count of usable conductance states.

**Figure S20. Number of effective conductance states as a function of strain.** Number of effective conductance states extracted from the *ΔG* distributions in Figure S19 using threshold*_ΔG_* = 0.3% of the conductance window. The number decreases monotonically with increasing strain (96, 91, 74 and 66 states at 0%, 10%, 20% and 30% strain, respectively), reflecting the increased weight-update nonlinearity and the narrowing of the usable conductance window under mechanical deformation.

**Figure S21. Recognition accuracy of the elastic dual-gate OECT-based artificial synapse under different pulse-training conditions. a)** Training accuracy of the artificial neural network as a function of epoch using conductance modulation characteristics with different LTP pulse amplitudes while the LTD pulse amplitude and pulse frequency are fixed. The inset compares the recognition accuracy at 125 epochs. **b)** Training accuracy of the artificial neural network as a function of epoch using conductance modulation characteristics with different LTD pulse amplitudes while the LTP pulse amplitude and pulse frequency are fixed. The inset compares the recognition accuracy at 125 epochs. **c)** Training accuracy of the artificial neural network as a function of epoch using conductance modulation characteristics with different pulse frequency while both LTP and LTD pulse amplitudes are fixed. The inset compares the recognition accuracy at 125 epochs.

**Supplementary Tables**

**Table S1. Comparison of our intrinsically stretchable OECT with other state-of-the-art OECT platforms in the literature, in terms of materials, stretchability, and volatile/non-volatile functionalities.**

**Table S2. Extracted equivalent circuit parameters obtained from electrochemical impedance spectroscopy (EIS) fitting for pure P(g2T2-T) and SEBS-blended P(g2T2-T) (PSES) films.** The fitting was performed using the equivalent circuit model shown in Figure S10c. The parameters include capacitance (C), Warburg element (W), charge transfer resistance (R_ct_), and series resistance (R_s_), reflecting charge storage and ion transport characteristics of the films.

**Table S3. Properties of the elastic dual-gate OECT-based artificial synapse under different pulse conditions.**

**Table S4. Properties of the elastic dual-gate OECT-based artificial synapse under different strains.**

**Table S5. Comparison of the elastic dual-gate OECT-based artificial synapse and other synaptic devices in terms of learning accuracy predicted by NeuroSim+ MNIST Multilayer Perceptron (MLP) V3.0 simulator.**

**Supplementary References**

[S1] S. Wang, X. Chen, C. Zhao et al., “An Organic Electrochemical Transistor for Multi-Modal Sensing, Memory and Processing,” *Nature Electronics* 6 (2023): 281–291, https://doi.org/10.1038/s41928-023-00950-y

[S2] X. Ji, B. D. Paulsen, G.K.K. Chik et al., “Mimicking Associative Learning Using an Ion-Trapping Non-Volatile Synaptic Organic Electrochemical Transistor,” *Nature Communications* 12 (2021): 2480, https://doi.org/10.1038/s41467-021-22680-5

[S3] Y. Dai, S. Dai, N. Li et al., “Stretchable Redox-Active Semiconducting Polymers for High-Performance Organic Electrochemical Transistors,” *Advanced Materials* 34 (2022): 2201178, <https://doi.org/10.1002/adma.202201178>

[S4] H. Jung, D. Lee, K. Kim et al., “Hydrogel–Elastomer-Based Conductive Nanomembranes for Soft Bioelectronics,” *Nature Nanotechnology* 20 (2025): 1822–1830, https://doi.org/10.1038/s41565-025-02031-x

[S5] S. Lu, J. Wu, B. Zhang et al., “Intrinsically stretchable vertical organic electrochemical transistor featuring sub-nanometer-roughness electrodes for high transconductance and stretchability,” *Nano Letters* 26 (2026): 3956–3966, https://doi.org/10.1021/acs.nanolett.6c00353

[S6] S. Dai, Y. Dai, Z. Zhao et al., “Intrinsically Stretchable Neuromorphic Devices for On-Body Processing of Health Data with Artificial Intelligence,” *Matter* 5 (2022): 3375–3390, https://doi.org/10.1016/j.matt.2022.07.016

[S7] H. Shim, S. Jang, A. Thukral et al., “Artificial Neuromorphic Cognitive Skins Based on Distributed Biaxially Stretchable Elastomeric Synaptic Transistors,” *Proceedings of the National Academy of Sciences* 119 (2022): e2204852119, https://doi.org/10.1073/pnas.2204852119

[S8] S. H. Jo, T. Chang, I. Ebong et al., “Nanoscale Memristor Device as Synapse in Neuromorphic Systems,” *Nano Letters* 10 (2010): 1297–1301, https://doi.org/ 10.1021/nl904092h

[S9] Y.-F. Wang, Y.-C. Lin, I-T. Wang et al., “Characterization and Modeling of Nonfilamentary Ta/TaO_x_/TiO_2_/Ti Analog Synaptic Device,” *Scientific Reports* 5 (2015): 10150. https://doi.org/10.1038/srep10150

[S10] S. Park, A. Sheri, J. Kim et al., "Neuromorphic Speech Systems using Advanced ReRAM-based Synapse," *IEEE International Electron Devices Meeting* (2013), https://doi.org/10.1109/IEDM.2013.6724692

[S11] J. Woo, K. Moon, J. Song et al., “Improved Synaptic Behavior Under Identical Pulses Using AlO_x_/HfO_2_ Bilayer RRAM Array for Neuromorphic Systems,” *IEEE Electron Device Letters* 37 (2016): 994, https://doi.org/10.1109/LED.2016.2582859

[S12] D. Kuzum, R. G. D. Jeyasingh, B. Lee et al., “Nanoelectronic Programmable Synapses Based on Phase Change Materials for Brain-Inspired Computing,” *Nano Letters* 12 (2011) 2179–2186, https://doi.org/10.1021/nl201040y

[S13] S. Choi, S. H. Tan, Z. Li et al., “SiGe Epitaxial Memory for Neuromorphic Computing with Reproducible High Performance based on Engineered Dislocations,” *Nature Materials* 17 (2018): 335–340, https://doi.org/10.1038/s41563-017-0001-5.

[S14] M. Jerry, P.-Y. Chen, J. Zhang et al., “Ferroelectric FET Analog Synapse for Acceleration of Deep Neural Network Training,” *IEEE International Electron Devices* Meeting (2017), https://doi.org/10.1109/IEDM.2017.8268338.
